# Stereotactic Radiosurgery in the Management of Patients With Brain Metastases of Non-Small Cell Lung Cancer: Indications, Decision Tools and Future Directions

**DOI:** 10.3389/fonc.2018.00154

**Published:** 2018-05-09

**Authors:** Dianne Hartgerink, Britt van der Heijden, Dirk De Ruysscher, Alida Postma, Linda Ackermans, Ann Hoeben, Monique Anten, Philippe Lambin, Karin Terhaag, Arthur Jochems, Andre Dekker, Janna Schoenmaekers, Lizza Hendriks, Jaap Zindler

**Affiliations:** ^1^Department of Radiation Oncology (MAASTRO Clinic), GROW School for Oncology and Developmental Biology, Maastricht University Medical Centre, Maastricht, Netherlands; ^2^Proton Therapy Department South-East Netherlands (ZON-PTC), Maastricht, Netherlands; ^3^Department of Radiology, Maastricht University Medical Centre, Maastricht, Netherlands; ^4^Department of Neurosurgery, Maastricht University Medical Centre, Maastricht, Netherlands; ^5^Department of Medical Oncology, Maastricht University Medical Centre, Maastricht, Netherlands; ^6^Department of Neurology, Maastricht University Medical Centre, Maastricht, Netherlands; ^7^Department of Pulmonary Diseases, Maastricht University Medical Centre, Maastricht, Netherlands

**Keywords:** brain metastases, non-small cell lung cancer, stereotactic radiosurgery, isotoxic dose prescription, shared decision

## Abstract

Brain metastases (BM) frequently occur in non-small cell lung cancer (NSCLC) patients. Most patients with BM have a limited life expectancy, measured in months. Selected patients may experience a very long progression-free survival, for example, patients with a targetable driver mutation. Traditionally, whole-brain radiotherapy (WBRT) has been the cornerstone of the treatment, but its indication is a matter of debate. A randomized trial has shown that for patients with a poor prognosis, WBRT does not add quality of life (QoL) nor survival over the best supportive care. In recent decades, stereotactic radiosurgery (SRS) has become an attractive non-invasive treatment for patients with BM. Only the BM is irradiated to an ablative dose, sparing healthy brain tissue. Intracranial recurrence rates decrease when WBRT is administered following SRS or resection but does not improve overall survival and comes at the expense of neurocognitive function and QoL. The downside of SRS compared with WBRT is a risk of radionecrosis (RN) and a higher risk of developing new BM during follow-up. Currently, SRS is an established treatment for patients with a maximum of four BM. Several promising strategies are currently being investigated to further improve the indication and outcome of SRS for patients with BM: the effectivity and safety of SRS in patients with more than four BM, combining SRS with systemic therapy such as targeted agents or immunotherapy, shared decision-making with SRS as a treatment option, and individualized isotoxic dose prescription to mitigate the risk of RN and further enhance local control probability of SRS. This review discusses the current indications of SRS and future directions of treatment for patients with BM of NSCLC with focus on the value of SRS.

## Introduction

Brain metastases (BM) are the most frequent intracranial malignancies and originate mainly from lung cancer (1). In patients with driver mutations of non-small cell lung cancer (NSCLC), systemic therapies have become more effective in patients with metastatic disease, resulting in longer overall survival (OS). Due to the screening for BM, the longer OS, and the often poor drug penetration through the blood–brain barrier (BBB), more and more patients are diagnosed with BM. BM may cause neurologic symptoms, a decrease in quality of life (QoL), and are often associated with poor OS (2).

Overall, patients with BM are treated with the intention to maintain QoL during their remaining lifespan. Traditionally, the treatment consists predominately of radiotherapy, mainly whole-brain radiotherapy (WBRT), but in selected patients, surgery, systemic therapy, or a combination of treatment modalities is used. Depending on the prognostic subgroups, the OS after WBRT in patients with BM of NSCLC without systemic treatment remains poor with an estimated survival of weeks or months (3–5). For instance, for patients treated with WBRT and optimal supportive care in the QUARTZ trial, the median survival was 8.5 weeks, and there was no OS benefit (4). Physicians are increasingly reluctant in the use of WBRT, as the results of the QUARTZ trial did not show a benefit of WBRT in NSCLC patients over steroids alone in patients with an intermediate or unfavorable prognosis [recursive partitioning analysis (RPA 2–3)] (4).

It is increasingly important to accurately estimate the prognosis after all treatment options, to support decision-making of both patients and physicians. OS of patients with BM of NSCLC ranges from several weeks to several years depending on relevant prognostic factors, such as performance status, age, control of extracranial disease, number of BM, and the presence of driver mutations. Gaspar et al. published a report in 1997 on a prognostic index for patients with BM, the RPA based on patients mainly treated with WBRT. The RPA was externally validated, and the favorable prognostic RPA score had a median survival of 7.1 months. The unfavorable RPA score had a survival of only 2.3 months (6, 7). The weakness of the RPA score is that the majority of the patients are classified into the intermediate prognostic class, and for clinical decision-making the favorable and unfavorable prognostic classes are the most important ones Also, the RPA score was developed in the pre-immunotherapy era, and cancers other than NSCLC were included into this score which limits its utility in patients with BM of NSCLC. Therefore, the Graded Prognostic Assessment (GPA) was developed from a database of almost 2,000 patients with BM, validated and refined with diagnosis-specific (DS) indices based on a second retrospective analysis of 4,259 patients with BM (8–10). For patients with BM of NSCLC and an unfavorable DS GPA score, the median survival time is 3 vs 15 months for patients with a favorable DS-GPA score (11). Recently, a refined Lung-molGPA score was developed and validated specifically for patients with BM of NSCLC. molGPA integrates molecular features such as epidermal growth factor receptor (EGFR) and anaplastic lymphoma kinase (ALK) alterations (12, 13). The overall median survival for the cohort was 12 months, and those patients with a Lung-molGPA score of 3.5–4.0 had a median survival of almost 4 years. It is for these reasons that lung-moGPA is the most useful prognostic tool for clinical practice in the era of personalized medicine and targeted agents.

In the last decades, a local alternative treatment became widely available, stereotactic radiosurgery (SRS). SRS has the advantage of achieving higher local tumor control while sparing healthy brain tissue, which results in less severe side effects such as neurocognitive damage and hair loss (14). SRS is currently a well-established treatment modality for patients with a maximum of four BM. SRS is also a more complex and costly treatment which may not be available in every radiotherapy department. Other disadvantages of SRS compared with WBRT are a higher risk of new BM during follow-up (e.g., distant brain recurrences), and an increased risk of radionecrosis (RN) depending on the volume of healthy brain tissue which is irradiated to a relatively high dose, tumor biology factors, and the location of the tumor. RN is focal damage of the nearby brain tissue caused by a high dose of radiation. The result of RN can be temporary or permanent neurologic symptoms. These symptoms can be treated with steroids, but steroids have several side effects such as obesity, sleeping disorders, hyperglycemia, and muscle weakness (15–17). The risk of RN is mainly correlated with the SRS dose in the brain and the size of the lesion, for example, if the volume of brain tissue which receives ≥12 Gy is more than 10 cm^3^, the risk of RN increases to above 10% and can go as high as 25%. However, other factors may contribute. The risk of distant brain recurrences is mainly correlated with the number of SRS treated BM and varies between 40 and 90% if SRS is applied as a single treatment modality (14, 18). From all patients treated with SRS as a single treatment modality for a maximum of three BM, 25% of patients will receive WBRT, because a significant proportion of patients die from extra cranial disease progression. It should be stated that almost all long-term survivors will undergo WBRT at some time point (14, 19, 20).

For small BM, SRS is equally effective as surgical resection (21). SRS is typically delivered in one to five fractions of multiple photon beams, but more recently even multiple, i.e., more than four, BM can be treated with a single fraction (22). For patients with more than four BM, the current Dutch guidelines advises WBRT as standard of care, but trials are ongoing and already conducted to investigate the value of SRS in patients with more than three BM (23–26).

The aim of this review is to discuss the current evidence of SRS for BM of NSCLC, potential improvements in patient information with focus on shared decision-making (SDM), and promising future treatment strategies to improve the clinical outcome.

## Current Evidence for SRS as a Single Treatment Modality

As mentioned previously, the indication of WBRT is currently a matter of debate: according to the QUARTZ trial, there was no beneficial effect of WBRT over steroids, in NSCLC patients, most with an RPA class 2–3, with respect to QoL or survival (4). There are several remarks with respect to clinical application of the results: patients were only included if the patient or the multidisciplinary team had doubts if WBRT should be applied, favorable prognostic patients (RPA class I) did have a survival benefit of WBRT, and the effect on symptom control such as seizures or headache were not described in detail. The latter is relevant, because the reason for applying WBRT is sometimes symptom control, prevention of neurologic symptom progression, or prevention of dying due to a neurologic cause. The availability of drugs used to target different areas for these patients is constantly increasing which may influence their outcome. Taking into account the limitations of the study which is conducted in a poor prognostic population, physicians should be more reluctant to apply WBRT (RPA class 3).

As an alternative to SRS, patients with small asymptomatic BM of NSCLC can be treated with systemic therapy (chemotherapy, targeted therapy, or immunotherapy in the second line). Targeted therapy can be considered if there is a driver mutation (27, 28). Cranial radiation can be considered if a low response rate is to be expected from systemic treatment or if neurological symptoms are to be expected in the event of disease progression.

Nowadays, the preference for either SRS or WBRT depends on the size and number of BM, but only if patients are in a good physical condition (Karnofsky performance status 70 or more). Patients with a maximum of four BM are usually treated with SRS, and patients with more than four BM are treated with WBRT. The size and location of the lesions are decisive factors: a very large BM with a diameter of 6 cm is inappropriate for treatment with SRS or a large brainstem metastasis, whereas five minor lesions located in the cerebral hemispheres are technically less challenging for treatment with SRS. In patients with a poor performance status (Karnofsky performance status of less than 70) are usually treated with supportive care. Patients treated with SRS despite a poor physical condition still have a very poor prognosis with a median survival of around 3 months (2).

The treatment of patients with asymptomatic BM from non-squamous NSCLC depends on molecular diagnostics, but primary systemic treatment with deferral of radiotherapy is a treatment option that could be considered (29, 30). For NSCLC, Lim et al. randomized 105 patients with 1–4 asymptomatic BM to receive SRS followed by chemotherapy or upfront chemotherapy alone (31). The trial closed early due to slow recruitment and was therefore underpowered. SRS followed by chemotherapy did not improve the OS compared with upfront chemotherapy (14.6 vs 15.3 months, *p* = 0.418). The time to central nervous system (CNS) progression was not significant different between the two arms [9.4 months (SRS) vs 6.6 months (upfront chemotherapy), *p* = 0.248].

Also for patients with asymptomatic BM SRS is an attractive first line of treatment. Approximately 25% of the patients with an EGFR-mutated NSCLC have BM at first presentation. For patients with an EGFR targetable mutated NSCLC, an alternative first line of treatment consists of an EGFR inhibitor like erlotinib, gefitinib, osimertinib, or afatinib with a response rate of approximately 60–70% (30, 32). Only a small percentage passes through the BBB and the penetration is different between the treatment options whereas osimertinib has a greater penetration. However, the response probability is equal to the extra-cerebral response probability (33, 34). Approximately 24% of ALK translocated NSCLC patients have BM at presentation (35). In case of an ALK translocation the treatment consists of alectinib, ceritinib, or crizotinib, with an expected response rate of approximately 50–60%, whereas alectinib has a superior CNS activity compared with crizotinib (36). If cerebral progression occurs during treatment with an EGFR or ALK inhibitor, SRS is considered, or a second line of systemic therapy. A recent study provides evidence for upfront SRS in patients with asymptomatic BM. Patients who are tyrosine kinase inhibitors (TKI) naïve and have an EGFR mutation had a better survival than patients who were treated with primary systemic therapy (37). This study was limited by its retrospective nature. Prospective randomized studies are needed to investigate if upfront SRS without TKI, directly followed by TKI or even concurrent, is beneficial over primary systemic treatment without SRS in TKI naïve patients.

### SRS for a Maximum of Four BM

The definition of limited BM traditionally consisted of patients presenting with a single BM, often treated with surgery. This definition has evolved to encompass patients presenting with up to three metastasis for treatment with SRS (38–41). The management of patients presenting with a limited number of BM and a good performance status has developed from WBRT alone to a more aggressive approach consisting of WBRT in combination with SRS (41–43). The necessity of WBRT was evaluated *via* clinical trials, which compared SRS alone to SRS with adjuvant WBRT (44–48).

Aoyama et al. reported the first randomized control trial comparing SRS alone with SRS plus WBRT, randomizing 132 patients with 1–4 BM from histologically confirmed systemic cancer, mainly NSCLC (67%) (46). The primary endpoint was cranial recurrence. Although the 1-year local control rate was higher in the SRS plus WBRT group (88.7 vs 72.5%, *p* = 0.002), there was no OS difference between the two study arms (trial was underpowered for the secondary endpoint survival). There was also no advantage with respect to cognition based on Mini-Mental Status Exam (MMSE) for patients receiving SRS plus WBRT. It should be taken into account that the MMSE is a crude measurement for neurocognition compared with a standardized test such as the Hopkins Verbal Learning Test (HVLT) or other neurocognitive tests proposed by the European Organization for Research and Treatment of Cancer (EORTC), for example (49–51). A secondary analysis of the JROSG 99-1 randomized clinical trial investigated the feasibility of SRS alone for patients with different prognoses defined by the DS-GPA (24). Significantly better OS was observed in the DS-GPA favorable group (scores 2.5–4.0) in WBRT + SRS vs SRS alone. However, it is questionable if the side effects of adding WBRT to SRS, justify a potential limited survival benefit in favorable prognostic patients.

Chang et al. also randomized patients into SRS alone or SRS plus WBRT treatment arms, but they took a different approach by evaluating patients’ neurocognition using the HVLT-revised (HVLT-R) as the primary endpoint (47). Patients presenting with 1–3 BM from different primary cancers, mainly NSCLC (55%), were randomized, 30 patients to SRS alone and 28 to SRS plus WBRT. The trial was prematurely stopped because at the interim analysis, patients in the SRS plus WBRT arm were more likely to have a decline in neurocognitive function 4 months posttreatment. They found an unexpected survival advantage (secondary endpoint) in the SRS alone arm, with an OS of 15.2 vs 5.7 months in the in the SRS alone, and SRS plus WBRT arms, respectively. The reasons for higher survival rates in the SRS alone arm were unclear. It may be explained by a higher rate of salvage cranial treatment. Moreover, chemotherapy was administered to more patients with a longer duration in the SRS group compared with the SRS plus WBRT group (52). The authors concluded that the management for patients presenting with one to three BM with SRS alone is the optimal treatment.

The EORTC evaluated SRS alone vs SRS plus WBRT with the primary endpoint of functional outcomes, using the World Health Organization (WHO) performance status scale, in patients with one to three BM from mainly NSCLC (53%) (44). They concluded that WBRT did not improve the duration of functional independence (WHO ≤ 2, SRS alone 10.0 months vs SRS plus WBRT 9.5 months). SRS plus WBRT reduced the incidence of radiological endpoints, such as distant brain failure and 2-year local control failure rate (radiosurgery group: 31–19%, *p* = 0.040). Despite the secondary outcomes, the QoL was worse in several domains for patients who received WBRT (31, 44). A secondary analysis of the EORTC 22952-26001 trial investigated the impact of WBRT on patients with a favorable GPA prognostic score. The primary endpoint was OS (45). There was no significant survival benefit for NSCLC patients with a favorable GPA score of WBRT + SRS over SRS alone. There was also no survival benefit for patients with controlled extracranial disease. This secondary analysis supports the practice of treatment with SRS alone for patient with limited BM.

Recently, Brown et al. reported the results of the North Coast Cancer Treatment Group (NCCTG) phase III study in patients with one to three BM, mainly from lung cancer, treated with SRS alone or SRS plus WBRT (53). Regarding the primary endpoint of neurocognitive function, the trial is comparable with Chang et al., except that Brown et al. randomized 208 patients (47). Cognitive progression, defined as a decline of >1 SD from baseline on ≥1 of the cognitive tests 3 months post-SRS, was higher in the SRS plus WBRT arm compared with SRS alone (91.7 vs 63.5%, *p* < 0.001), and cognitive deterioration was more frequent in long-term survivors (living 12 months or more) receiving WBRT plus SRS compared with SRS alone (94.4 vs 60%). The 1-year intracranial disease control was 50.5% in the SRS alone arm and almost 85% in the SRS plus WBRT arm. The secondary survival analysis showed a median OS of 10.4 months for SRS alone vs 7.4 months for SRS plus WBRT. The authors concluded that SRS alone is preferred for patients presenting with one to three BM, supporting the results of Chang et al.

A secondary analysis of the NCCTG randomized control trial from Brown et al. was performed by Churilla et al. to determine whether WBRT is associated with improved OS among NSCLC patients with favorable prognoses (DS-GPA ≥ 2.0 or ≥2.5) at diagnosis (53, 54). They used two separate cut-points of DS-GPA, ≥2.0 vs <2.0 and ≥2.5 vs <2.5 in a study population consisting of 126 NSCLC patients with 1–3 BM. For patients with DS-GPA ≥ 2.0 treated with SRS alone, the median survival was 17.9 vs 11.3 months in the SRS plus WBRT arm (*p* = 0.63), and 6.6 vs 3.7 months for patients with DS-GPA < 2.0 (*p* = 0.85). They observed no significant differences in survival analysis in favorable-prognosis NSCLC patients treated with SRS, with or without WBRT, which further supports the approach of SRS alone. The above trials, summarized in Table 1, demonstrate that adjuvant WBRT results in reduced QoL and neurocognitive function without improvement of OS.

**Table 1:** Summary of selected trials evaluating the role of SRS ± WBRT for patients with limited brain metastases.

Trial	Patient selection	Primary endpoint	Local control	OS	Functional outcome
Aoyama et al. (46) SRS *N* = 67 WBRT + SRS *N* = 65	1–4 metastases, KPS ≥ 70, lesion diameter <3 cm	Cranial recurrence	1 year: 72.5 vs 88.7% (*p* = 0.002)	1 year: 28.4 vs 38.5% (*p* = 0.42)	No difference in cognition based on MMSE

Aoyama et al. (24) SRS *N* = 45 WBRT + SRS *N* = 43	1–4 metastases, NSCLC patients	OS according DS-GPA score	–	DS-GPA favorable: 10.6 vs 16.7 months (*p* = 0.04) DS-GPA unfavorable: 6.5 vs 4.75 months	No difference in neurocognitive function based on MMSE

Chang et al. (47) SRS *N* = 30 WBRT + SRS *N* = 28	1–3 metastases, KPS ≥ 70	Neurocognition (using HVLT-R)	1 year: 67 vs 100% (*p* = 0.012)	15.2 vs 5.7 months	HVLT-R decline 52 vs 24%

Kocher et al. (44) SRS *N* = 100 WBRT + SRS *N* = 99	1–3 metastases, WHO ≤ 2	Functional independence (WHO ≥ 2)	2 year: 69 vs 81% (*p* = 0.04)	10.9 vs 10.7 months (*p* = 0.89)	No difference 10.0 vs 9.5 months

Brown et al. (51) SRS *N* = 111 WBRT + SRS *N* = 102	1–3 metastases, diameter < 3 cm, ECOG performance score ≤2	Cognitive deterioration	3 months: 75.3 vs 93.7% (*p*< 0.001)	10.4 vs 7.4 months (*p* = 0.92)	Higher deterioration in verbal fluency and delayed/immediate memory in SRS + WBRT arm

Churilla et al. (53) SRS WBRT + SRS	1–3 metastases, NSCLC patients	OS according DS-GPA score	–	10.8 vs 7.5 months	No difference in survival in favorable-prognosis NSCLC patient

*KPS, Karnofsky performance status; WBRT, whole-brain radiotherapy; SRS, stereotactic radiosurgery; WHO, World Health Organization; HVLT-R, Hopkins Verbal Learning Test revised; OS, overall survival. NSCLC, non-small cell lung cancer. MMSE, Mini-Mental State Examination; DS-GPA; diagnosis-specific Graded Prognostic Assessment*.

The current available evidence supports the use of SRS as a single treatment modality in patients with a maximum of three BM. This is supported by the American Society for Therapeutic Radiation Oncology (ASTRO).

### SRS for More Than Four BM

For patients presenting with >4 metastases, traditionally WBRT has been the standard of care. In patients with >4 metastases, the application of SRS is controversial, because the currently discussed randomized trials were done in patients with a limited number of BM. The additional palliative value of SRS over WBRT in patients with four or more BM remains to be determined. An international practice survey reported in 2010 showed the difference in consensus on SRS. In a responder-population consisting of SRS-specialists, 83% would consider patients >4 brain metastasis as potential candidate for SRS. By contrast, only 7% of a responder-population consisting of radiation oncologists agreed for SRS in patients with >4 BM (55). Physicians that support SRS in patients with >4 metastases indicate that in the past there where technical issues concerning the long treatment times and safety of the radiation doses (56–59).

The multi-institutional prospective study from Yamamoto et al. was the first evaluating SRS alone for four and more BM (22). The trial population consisted of favorable prognostic patients with low volume BM, three-quarters originate from primary lung cancer, the majority had an RPA 2 and KPS ≥ 80 (largest tumor <10 mL in volume, <3 cm in longest diameter; total cumulative volume ≤15 mL). This study included 1,194 patients with 1–10 metastases and where treated with SRS, split in to three cohorts: 208 patients with 5–10 metastases, 531 patients with 2–4 metastases, and 455 patients with a single metastasis. The intention was to determine non-inferiority in the cohort with 5–10 BM compared with 2–4 BM with OS as the primary endpoint. The OS did not differ between patients with 5–10 BM or 2–4 BM (HR 0.97, *p* = 0.78). As well as local control rates, distant brain relapses were not significantly different between both cohorts. This suggests that SRS without WBRT in patients with 5–10 BM is non-inferior to patients with 2–4 BM. SRS may be an alternative for WBRT as SRS is minimally invasive and has fewer side effects.

A second case-matched, retrospective cohort trial from Yamamoto et al. compared treatment results for patients with 10 or more BM vs 2–9 metastases (60). The primary endpoint was OS, whereas the secondary endpoints consisted of neurological death and deterioration, local recurrence and repeat SRS, and major complications of SRS. The median survival time between the two arms did not differ significantly, 6.8 months for patients with 2–9 BM vs 6.0 months for patients ≥10 BM (*p* = 0.10). Considering the incidence of neurological deterioration (defined as any brain disease-caused neurological worsening), there was no difference between the groups, including radiation-related complications. They concluded that treatment results after SRS were not inferior for patients with 10 or more BM than for patients with 2–9 metastases.

Serizawa et al. conducted a small retrospective study to compare the effectiveness of SRS (*N* = 62) with WBRT (*N* = 34) for multiple cranial metastases from non-small-cell lung cancer (61). They included patients with 1–10 BM with a life expectancy of more than 2 months, lesions >3 cm were surgically removed. The OS time and the neurological survival in the SRS arm were significantly longer. The risk of neurologically impaired QoL was also lower in the SRS arm.

The results of these studies support the hypothesis that SRS is a viable treatment option in patients with four or more BM. The main question if SRS is beneficial over WBRT with respect to QoL, survival, and maintenance of neurocognitive function, needs to be answered in randomized trials (25).

There are no published randomized trials evaluating the role of SRS in patients with ≥4 BM. In the Netherlands a randomized phase III trial (NCT02353000, https://clinicaltrials.gov/show/NCT02353000) is enrolling patients with 4–10 BM, KPS ≥ 70, and any primary solid tumor including NSCLC. The standard treatment WBRT is compared with SRS for all lesions, with the primary endpoint being of QoL at 3 months after radiotherapy (25). Another randomized phase III trial (NCT01592968) at the MD Anderson Cancer Center is randomizing patients with 4–15 BM to SRS alone vs WBRT alone. The primary endpoints are cognitive function and local tumor control at 4 months. If these trials are successful, SRS will replace WBRT as the standard treatment for patients with more than four BM. Several other trials are currently being initiated to evaluate the role of SRS in patients with multiple BM, such as Heidelberg (NCT0329778) and Boston (NTC03075072), among others.

**Figure 1:**
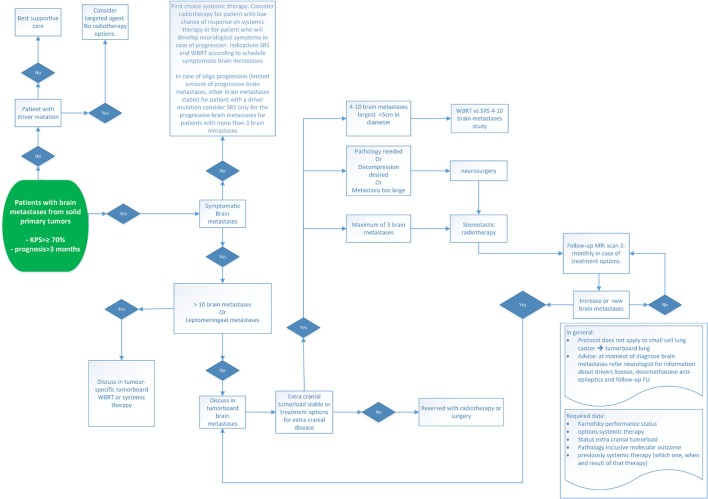
Treatment options for BM of NSCLC. Abbreviations: KPS, Karnofsky performance status; WBRT, whole-brain radiotherapy; SRS, stereotactic radiosurgery; BM, brain metastases; MRI, magnetic resonance imaging; NSCLC, non-small cell lung cancer.

Another treatment option for patients with multiple BM, especially in patients with asymptomatic BM of a driver mutation, is to only treat the larger and worrisome BM with SRS (Figure 1). This strategy is of specific interest in subgroups of patients who may survive over several years due in part to several lines of targeted agents and to postpone the radiotherapy (either SRS or WBRT) until progression of intracranial disease.

## Improving Indication of Cranial Irradiation as a Treatment Option for Patients with BM During SDM

Especially in the palliative setting of BM, it will be increasingly important to better inform the patient about the available treatment options, such as SRS, to individualize the multimodality treatment of patients with BM of NSCLC.

Shared decision-making is based on the principle of the person’s autonomy and to improve the participation of patients in making decision concerning their personal health and treatment. It can be divided into four parts: the health-care professional needs to inform the patient that a decision needs to be made and that the opinion of the patient also important is, the explanation from the health-care professional to the patient about the different options inclusive of advantages and disadvantages of the different treatment options, a discussion between the health-care professional and the patient about their preferences, and finally to make a decision about the chosen treatment (62).

Traditionally, physicians had a more paternalistic approach with respect to treatment choices based on national guidelines. However, for patients with BM with incurable cancer, it is of specific relevance to consciously make choices between treatment options taking into account all advantages and disadvantages. With the availability of an increasing number of treatment options, SDM is challenging and complex, and work is in progress to make SDM tools available for the patient and caretakers.

### Decision Support Systems (DSSs) Including SRS as a Treatment Option

A DSS is a direct aid in clinical decision-making, in which characteristics of individual patients are used to generate patient-specific assessments or recommendations that are then presented to clinicians for consideration (63).

In radiotherapy oncology, the DSSs use advanced and innovative information technology, combined with available medical data to achieve the highest possible accuracy in the prediction of everything from the response of the tumor, to the treatment response and toxicity of normal tissue (64). The base of SDM is individualized prognostic models for outcome prediction per patient. Individualized prognostic models use machine learning algorithms to learn from patients treated in the past to make predictions for patients we currently see in the clinic. Traditionally, outcome was calculated per group of patients, such as the RPA (65).

Recently, individualized prognostic models became available for patients treated with SRS for a limited number of BM of NSCLC (66). These two models can predict individualized chances of both early death (<3 months), and long-term survival (>12 months), after SRS for BM of NSCLC, with an area under the curve (AUC) for early death of 0.79 with a 95% confidence interval (CI) of 0.72–0.86, and an AUC for long-term survival of 0.77 with a 95% CI of 0.70–0.84. The nomograms were more accurate in the prediction of early death and long-term survival than commonly used prognostic scores after SRS for a limited number of brain metastasis of NSCLC. Examples of commonly used prognostic scores are the RPA, the Golden Grading System, DS-GPA, and Score Index for Radiosurgery in brain metastasis (SIR) (66). Other than the increased accuracy of prediction, these nomograms are also easy to use in routine clinical practice and are available at www.predictcancer.org (66). Other tumor-specific individualized models are necessary for more relevant clinical endpoints, such as the occurrence of distant brain recurrences, local control probability, and the risk of RN or cognitive toxicity. The current individualized prognostic models are relatively simple and may become more accurate in outcome prediction if more patient-, treatment-, and tumor characteristics are added into these models. With respect to the latter genomic and radiological factors with biomarkers, radiomics, and deep learning are of specific interest (64, 67–70). Model performance is in part, dependent on the volume of patient data used on which to base these models (71). Unfortunately, sharing of data between hospitals is hampered by ethical, political, administrative, and legal barriers (72). Efforts have been made to learn from multiple hospitals while avoiding the hurdles associated with sharing data. The EuroCAT project is an example of one such effort, in which the distributed learning approach is championed (see the animation at https://www.youtube.com/watch?v=nQpqMIuHyOk) (73). Distributed learning is defined as learning from data stored at the hospital without the data leaving the hospital. As only model coefficients are transmitted, patient privacy is preserved while the data can still be used for teaching models. Models for survival and treatment-related side effects have been learned using this approach (64, 73–75).

### Patient Decision Aids Including SRS as a Treatment Option

Patient decision aids are based on prognostic models which can be individually adapted to the characteristics of the patient and their disease. The model consists of two key steps: information exchange and deliberation between the health-care professional and the patient (76). The goal of these tools is to achieve an optimal individualized treatment strategy. The patient benefits of SDM have been proven with level 1 evidence on more than 30,000 patients, and yet clinical decision-making remains complex; patients must not only weigh several treatment options in terms of benefits and harms but also absorb a large amount of technical information while dealing with the emotional burden of their disease (25, 77). Lack of awareness about treatment options can lead patients to choose treatments that are more expensive or not aligned with their values. To improve patients’ QoL, health-care quality, and to reduce unnecessary procedures, it is crucial to empower patients with solid decision support. Evidence shows that patients who use decision aids are better informed about their treatment options, and experience less decisional conflict, and less anxiety about their care. Despite the evidence, patient decision aids are not routinely applied in practice (77). The shared decision tools are focused on the patient, which means that the level of these tools is also adjusted to the average patient. Medically complicated terms will be avoided as much as possible (78). An example of a potential patient decision aid is a tool which will be designed for SRS treatment in NSCLC patients consists of several headings: who is the main health-care professional during the treatment, what is the path during the diagnosis to brain metastasis, what makes the treatment eligible for the patient, what does the treatment consist of, what are the advantages of the treatment, what are the disadvantage of the treatment including possible adverse events, what is the influence of the treatment on the life of the patient, and what does the follow-up consist of. Ideally, every treatment option will be incorporated within this decision aid.

When the patient has run through this decision tool, the patient and the health-care professional will discuss this tool, and the patient has the opportunity to ask questions about the content and the information in this tool. After this discussion, the patient and the health-care professional will determine, together, if the SRS treatment is the best treatment option for this patient. The goal of this SDM is to obtain an optimal individualized treatment strategy by making use of the shared decision tool in which there is a deliberation between the health-care professional and the patient (78).

## Future Directions of SRS for BM of NSCLC to Further Improve Outcome

### Combining Systemic Therapy With SRS for BM of NSCLC

To further improve the clinical outcome of SRS in patients with BM of NSCLC, it can be considered to combine targeted therapies with either SRS or WBRT. The hypothesis is that systemic therapies have a superior penetration through the BBB after radiotherapy leading to a better response in the brain of the systemic agent. Considering multiple reports on the efficacy of targeted therapy on BM, it is interesting to investigate if the combination of SRS with systemic treatment improves efficacy above systemic treatment only (79, 80).

A few trials combined cranial radiation with targeted therapies in patients with BM from primary NSCLC. Lee et al. randomized 80 NSCLC patients with KPS ≥ 70 and multiple BM to erlotinib or placebo arms, both concurrent with WBRT (81). Patients continued with treatment of erlotinib or placebo until progression of disease. The neurological progression-free survival (nPFS) and median OS are not significantly different with an equal nPFS in both treatment arms of 1.6 (*p* = 0.84), and a median OS of 2.9 and 3.4 months in the placebo and erlotinib arms, respectively (*p* = 0.83). More grade 3/4 toxicity was found in the erlotinib arm compared with the placebo arm for fatigue and rash and there was no reported difference in the QoL. They concluded that there was no improvement of nPFS or OS for erlotinib concurrent to WBRT for patients with EGFR wild-type NSCLC and multiple BM.

A phase III trial from Sperduto et al. randomized 126 NSCLC patients with 1–3 BM into WBRT plus SRS vs WBRT plus SRS plus erlotinib or temozolomide (82). Erlotinib or temozolomide could be continued up to 6 months after radiation. The trial closed early because of slow accrual. There was no statistically difference between the groups concerning OS and time to CNS progression. The performance status was inferior in the group with erlotinib or temozolomide compared with the group treated with WBRT plus SRS. They found more grade 3–5 toxicity in the patients treated with targeted therapy concurrent with radiation. The trial did not support the concurrent use of systemic treatment with WBRT plus SRS. The question remains if SRS only combined with erlotinib or temozolomide provides benefit over either SRS or systemic treatment only, in these patients.

Another phase II trial from Welsh et al. enrolled patients with BM from NSCLC who received erlotinib concurrently with WBRT, followed by maintenance erlotinib (83). The overall radiologic response rate was 86% and there was no increase in neurotoxicity and no grade ≥4 toxicity. The median survival was 9.3 months for EGFR wild-type patients and 19.1 months for patients with an EGFR mutation. The addition of erlotinib to cranial radiation was well tolerated, but there was no control arm.

Magnuson et al. preformed a multi-institutional analysis with 351 EGFR-mutant NSCLC patients who developed BM with no prior treatment with EGFR-TKI (37). Patients received EGFR-TKI followed by SRS or WBRT at intracranial progression (*N* = 131), WBRT followed by EGFR-TKI (*N* = 120), or SRS followed by EGFR-TKI (*N* = 100). The analysis demonstrated that the use of upfront EGFR-TKI, and deferral or radiotherapy is associated with inferior OS. SRS followed by EGFR-TKI resulted in the longest OS (46 vs 25 months for the EGFR-TKI arm). The high effective doses of SRS may ablate BM, whereas systemic therapy simultaneously controls the extracranial diseases and possibly intracranial micro metastatic disease. A prospective trial comparing SRS followed by EGFR-TKI vs EGFR-TKI only is urgently needed.

Hendriks et al. published a systematic review to address the toxicity of combining cranial radiotherapy (SRS, WBRT, or SRS + WBRT) with TKI such as erlotinib and gefitinib, focusing on neurological toxicity (84). Fifteen articles were included, with only one phase III randomized trial. The authors concluded that there are arguments that TKI can safely be combined with WBRT, lacking high-level evidence. Grade 3 or higher toxicity may increase combining TKI with SRS and WBRT. The systematic review emphasizes the need for further investigation. Two retrospective studies analyzed patients with concurrent crizotinib, an ALK inhibitor, and SRS for cranial and extracranial metastatic NSCLC patients. They concluded that SRS can be used safely in patients receiving crizotinib. When SRS was administered to the patient, crizotinib could be longer admitted to the patient, leading to a longer 2-year OS (72% duration of crizotinib >12 months vs 12% when duration of crizotinib <12 months) (85, 86). For ALK-rearranged patients, minimal data are available, further high quality studies evaluating the use of ALK inhibitors concurrent with SRS are needed.

### Combining SRS With Immunotherapy

A potential radiobiological advantage of SRS is an enhanced antitumor immune response after radiation of the tumor as mediator of systemic effects, better known as the abscopal effect (87, 88). Radiotherapy changes the tumor environment resulting in the release of tumor antigens and therefore increases the antitumor effect of immunotherapy. For example, IL2 is a cytokine, which plays a role in the activation of the immune response against tumor cells. L19 targets the extra domain B of fibronectin, which is a marker for tumor neoangiogenesis and is, among others, overexpressed in NSCLC. L19 can significantly increase the immune response. Zegers et al. provide evidence for an increased therapeutic potential due to the combination of radiation therapy with L19-IL2 (89).

Reynders et al. published an overview of preclinical and clinical data about the abscopal effect, resulting in 37 articles. They found a median time to abscopal effect of 5 months, and an ongoing median time of abscopal response of 13 months before disease progression (88). The included data point toward a synergy between immune treatment and radiotherapy, but further research is needed before the abscopal effect can become relevant in clinical practice.

The abscopal effect is well discussed in the literature concerning patients with BM of metastatic melanoma. Schoenfeld et al. reviewed 16 patients with BM of melanoma treated with ipilimumab and cranial radiation to evaluate the abscopal effect. They found a superior OS for patients who received SRS before ipilimumab compared with patients receiving ipilimumab before SRS (26 vs 6 months, *p* < 0.001) (90). Also, Knisely et al. found an increased survival rate for patients with BM of melanoma treated with SRS in addition to ipilimumab, with a median survival of 21.3 months in the SRS plus ipilimumab group vs 4.9 months in the ipilimumab group and a 2-year survival of 47.2 vs 19.7% (91). Both trials suggesting a role for radiation therapy in enhancing immunotherapy.

This phenomenon was also demonstrated in a case report regarding a patient with metastatic lung adenocarcinoma treated with ipilimumab concurrent with radiation (92). A complete response was received at the initial site and all metastatic sites. Posttreatment, an increase in tumor-infiltrating cytotoxic lymphocytes and tumor regression was seen. They concluded that this approach in NSCLC may establish clinical trials for patients with advanced metastatic disease in the future. Combining immunotherapy with SRS in patients with BM of NSCLC to induce an abscopal effect and improve outcome is a strategy which is currently being investigated in clinical trials, NTC02086721, for example.

### Individualized Isotoxic Dose Prescription (IDP) With SRS for Avoidance of RN and Improvement of Local Control

A disadvantage of SRS is the risk of RN with current SRS dose prescription. The SRS dose is prescribed based on the size of the planning target volume (PTV) of the BM and ranges between 15 and 27 Gy in one or three fractions. In larger BM, the dose is hypofractionated and lowered to avoid the risk of RN. Despite this strategy, the risk of RN is still increased in BM with a diameter of more than 2 cm as the volume of healthy brain tissue which is irradiated to a high dose remains relatively high (93).

A relatively new possible strategy to mitigate the risk of RN and to increase the local control probability is *IDP* (94). The idea of IDP is to prescribe the dose based on the normal tissue tolerance level instead of the size of the PTV so that the risk of complications is always minimized or even avoided. The dose in the PTV is escalated until the highest dose which is technically achievable. If the local control probability is unsatisfactory for the patient, the number of fractions can be increased to compensate. In the literature fractionated stereotactic radiotherapy (FSRT), approaches have been described to improve the local control in large BM (95, 96). These studies use a fixed prescribed dose, for example, 25 Gy in five fractions. FSRT has the increased risk of observing RN with increasing size of the BM due to the fixed prescribed dose, while IDP has the advantage that the tolerance level of the healthy brain tissue is always respected. IDP will increase the local control probability of patients with a diameter of less than 2 cm compared with current SRS dose prescription and decrease the risk of RN in BM with a diameter of more than 2 cm. IDP, therefore has the potential to increase the therapeutic ratio, e.g., ratio local control/RN, for all sizes of BM (94). IDP is expected to yield the best results as the margins used are minimized or even avoided, with an optimal beam arrangement (non-coplanar vs coplanar SRS beams). Predictive studies for IDP have already been published, such as an *in silico* study for NSCLC patients (97). Individualized IDP, compared with conventionally prescribed fractionated radiotherapy, enabled a therapeutic gain in almost 80% of the patients. In a predictive modeling study, a 25% increase in the estimated tumor control probability was expected with IDP for patients with NSCLC (98). Nowadays IDP is not yet in active clinical use, clinical studies are needed to validate the results achieved with this *in silico* study.

## Conclusion

In recent years, the management of lung cancer has changed dramatically. At present, patients having NSCLC with driver mutations are treated with multiple lines of systemic therapy leading to an increasing importance of the management of BM. The indication of WBRT is a matter of debate because of its side effects and relatively poor outcome in terms of QoL and survival after treatment. SRS is an emerging strategy for patients with BM of NSCLC and the standard treatment for patients with a maximum of three BM. SRS is also a promising treatment option for patients with four or more BM and randomized trials are ongoing to determine its value. Promising future strategies include combining SRS with systemic treatments, for example, upfront TKIs to improve survival by destruction of the BBB and better penetration of SRS. The combination of SRS with immunotherapy is promising to induce an abscopal effect. Finally, a promising strategy is the potential improvement of outcome of SRS in large BM by individualized IDP. With this strategy, the risk of RN is minimized or even avoided with a simultaneous improvement of the local control probability.

## Author Contributions

DH and BH have written the manuscript with input from all the authors.

## Conflict of Interest Statement

MAASTRO Clinic has a research agreement with Varian Medical Systems, Palo Alto, CA, USA. DR: Advisory board of Merck/Pfizer, Roche/Genentech, Bristol Myers Squibb, Celgene. The reviewer SW and handling Editor declared their shared affiliation.
